# Genetic variation for growth and selection in adult plants of *Eucalyptus badjensis*

**DOI:** 10.1590/S1415-475738420150041

**Published:** 2015

**Authors:** Paulo Eduardo Telles dos Santos, Estefano Paludzyszyn, Lorenzo Teixeira de Melo da Silva, Paula Burigo Vandresen

**Affiliations:** 1 Embrapa Florestas, Colombo, PR, Brazil; 2 Celulose Irani S.A., Campina da Alegria, SC, Brazil

**Keywords:** eucalypt, progeny test, genetic parameters, breeding, genetic gain

## Abstract

The aim of this study was to evaluate *Eucalyptus badjensis* concerning the genetic variation for growth traits and the potential of the species in supporting a breeding programme. The field trial was a provenance/progeny test established in Campina da Alegria, Santa Catarina, Brazil (latitude 26°52′05.1″ S, longitude 51°48′47.5″ W, altitude 1,015 m) in a soil classified as Latossolic Alumino-Ferric Brown Nitosol. The experiment comprised 60 open-pollinated progenies from the provenances *Glenbog* and *Badja State Forest*, New South Wales, Australia. Ten replicates and plots with six plants in row were used. At the age of 17 years, 279 trees were assessed for diameter of the bole at breast height (DBH), total tree height (H) and volume of wood with bark (Vol). After submitting the data to statistical genetic analysis, the overall means for DBH, H and Vol were 45.17 cm, 33.30 m and 2.84 m^3^, and the estimates of additive coefficient of variation [${\hat{CV}}_{a}$(%)] were 12.59%, 5.91% and 26.51%, respectively. Heritability coefficients of additive effects (${\hat{h}}_{a}^{2}$) were also estimated and the following values were found: 0.443, 0.312 and 0.358. Thirty-nine trees from 25 different progenies were selected. The expected means of the provenances after improvement were 50.02 cm, 34.35 m and 3.47 m^3^ for DBH, H and Vol, respectively.

## Introduction

Eucalypt plantations in the southern region of Brazil represent slightly over 11% of the total area cultivated with this genus in the country, nowadays estimated at 5.47 million hectares (based on information from IBÁ, 2014). In this region, the wood harvested has been used predominantly by the pulp and paper industry, followed by the bioenergy sector, which is mainly represented by firewood and woodchip as sources of heat. The wood to attend energetic purposes has been used for drying grains prior to storage, generation of steam to attend industrial needs, and production of several items utilized by the building industry. A small amount has been transformed into charcoal for domestic consumption, the same being the case with firewood allocated to heat water, home environments and to prepare food. Particularly in subtropical climate zones, parts of the pine tree plantations have been periodically substituted by eucalypt, both by forest companies and independent wood producers, mainly as a response to economic factors. This should be considered an additional factor contributing to the increase of interest in eucalypt wood. In view of these circumstances, it has been necessary to make available improved germplasms in order to attend extra demands whenever required.

In Brazilian high-altitude fields of the Atlantic Forest biome and inner lands of the Pampa biome in which eucalypt commercial plantations are established, the main cause of productivity reduction and mortality is thermic stress, as a consequence of very low temperatures hitting the plants throughout the winter. The effect of this abiotic component differs in terms of intensity, depending on the level of cold tolerance of the germplasm, the altitude of the terrain, and the relief shape.

The eucalypt species traditionally planted in areas of southern Brazil prone to severe frosts (latitudes between the parallels 24° and 32° S and altitudes over 750 m) are *Eucalyptus benthamii* and *E. dunnii*. *E. viminalis* is also still planted, despite of its limitations in terms of silvicultural traits and wood quality for multiple uses. The fact is that, when facing the options of germplasms gathering the traits cold tolerance, feasibility of clonal propagation and multiple uses of the wood, a very small number of alternatives for planting are available in regions prone to strong intensity cold events. Based on this reality and taking into account the convincing performance during the last 15 years, especially in 2000, 2004, 2008, 2010 and 2013, in which absolute minimum temperatures of −12 °C were reported, the species *E. badjensis* emerged as a feasible alternative. Genetic improvement efforts devoted to this species are of great interest in order to increase the restricted number of options that are currently available.

One of the advantages of *E. badjensis* is the ease of sprouting, according to several visual observations made in Brazil and published reports from studies carried out in South Africa (Swain and Gardner, 2003; Little and Gardner, 2003). This ability represents a great advantage in short rotation regimes, associated with the method of simple coppice, as usually done in plantations intended for energetic purposes. Concerning its potential for pulp making, the species is seen as suitable (Swain and Gardner, 2003; Thompson, 2012), increasing the possibilities of usage of the wood obtained from plantations. For non-timber uses, a good potential has been observed in the exploitation of phenolic compounds and essential oil for pharmacological and industrial applications, with an output level of 4% of 1,8-cineol (content over 80%) in the hydro-distillation of dried leaves (Antônio RD, 2011, Masters Dissertation, Universidade Federal do Paraná, Curitiba, PR).

According to Boland *et al*. (1984), *Eucalyptus badjensis* is described as a species of very restricted geographical dispersion in Australia, and its natural populations are concentrated in the southeastern portion of the state of New South Wales, occupying ranges of only 36° to 36°45′ S of latitude and 800 to 1,200 m of altitude. In this region, the climate is sub-humid, with the mean maximum temperature of hottest month in the range of 22 °C to 25 °C and the mean minimum of the coldest month around −4 °C to 0 °C. Concerning frosts, the authors state that the number of events can be greater than 100 each year and some snowfalls can be expected annually as well. The mean annual precipitation is around 800 to 1,200 mm, with a relatively even distribution. Finally, in terms of soils, on the better sites the species can be found growing over Podsols, but the natural occurrence is predominantly on very strongly limited conditions, such as Lithosols, with poor differentiation into horizons and stony or gravelly permeated.

Based on the above description, it is possible to deduce that a significant part of the conditions present in Australia also occur in several locations of the southern portion of the Brazilian territory, where eucalypts have been planted, especially on high elevation sites located in the state of Santa Catarina. In these regions, the possibility of success in cultivating *E. badjensis* is, in theory, the same as for *E. benthamii*, which is the most common subtropical eucalypt species planted by private companies and rural farmers in very cold areas. Accordingly, Higa *et al*. (2002) reported that in the year 2000 strong frosts occurred in the state of Santa Catarina and, despite to this, no damages were detected in young leaves and sprouts of plants growing in the location of Campina da Alegria.

With the aim to evaluate the genetic improvement potential of *Eucalyptus badjensis* for growth rate, the present work was carried out in a provenance/progeny test established by the company Celulose Irani S.A. near Campina da Alegria, a village of the municipality of Vargem Bonita-SC. At the age of 17 years, genetic parameters, additive values of genotypes and genetic gains were estimated for growth traits.

## Materials and Methods

The provenance origins of *E. badjensis* evaluated in the present study are *Glenbog* (P1) and *Badja State Forest* (P2), New South Wales, Australia. The geographical and climatic features of both locations are summarized in Table 1. The geographical coordinates and climate from which the seeds were collected in Australia are comparable to some extent to those found in certain regions of southern Brazil, where eucalypts are commonly planted. This is particularly true with respect to the altitude and absolute minimum temperature. In contrast, the average number of frosts in Brazil is lower than in Australia and varies widely from one year to another.

**Table 1:** Details of the *E. badjensis* provenances assessed in Campina da Alegria-SC.

Origin of the provenance	N° of progenies	Latitude (S)	Longitude (E)	Altitude (m)	Soil type	Average number of frosts per year	Minimum absolute temperature
*Glenbog* (P1)	20	36°29′	149°19′	1,100	Red clayey loam	82	−12 °C
*Badja State Forest* (P2)	40	36°02′	149°34′	1,000	Reddish brown clayey loam	82	−12 °C

Source: datasheets of the supplier of *E. badjensis* seedlots to Brazil (Kylisa Seeds Pty Ltd.).

We admitted the degree of relatedness of half-sibs for the plants that compose each progeny, mainly due to the fact that they are the result of open-pollination process. This allowed us to adjust the most appropriate mathematical model for estimating the intended genetic parameters and other calculations that supported the present study. The field trial was established on March 19, 1996, following a compact family block design, with 10 replicates and linear plots containing six plants each. The plant-to-plant distance within rows was 2 m and the spacing between rows was 3 m. Two lines surrounding the trial composed the border, with the same layout. The total area of the experiment was 2.2 ha. At the time of assessment, the experiment had already been thinned two times, lowering the density of plants to approximately 165 individuals per hectare.

The geographical coordinates of the site in which the field trial was done are: latitude 26°52′05.1″ S, longitude 51°48′47.5″ W and altitude 1,020 m above sea level. According to W. Köppen's system, the climate type in Campina da Alegria-SC is classified as warm temperate (Cfb), with a great number of cold hours accumulated during the winter. With respect to precipitation, the mean annual reaches 2,030 mm (Climatic Station of Irani-SC), well distributed along the seasons and with occasional dry periods. There is always a positive difference between the monthly mean precipitation and the potential evapotranspiration (Wrege *et al*., 2011). The risk of frosts is high, because of the geographical position of the location associated with the expressive altitude (Santa Catarina, 1958). The relief of the region is typically slightly undulate to undulate. In a soil survey performed in 2013 by researchers of Embrapa, the soil of the site was classified as Latossolic Alumino-Ferric Brown Nitosol, showing a noticeable humic “A” horizon and a very clayey texture (J.B.V. Gomes and I.A. Bognola, personal communication).

Each tree was assessed at the age of 17 years for the following traits: diameter of the bole at breast height (DBH) in centimeters, total tree height (*H*) in meters and, by calculation, volume of wood with bark (Vol) in cubic meters. In order to compute the effective volume of wood, a form factor of 0.50 was adopted. For the determination itself, the following expression was used:

(1) $$\text{Vol}=\left(\frac{\text{π}\cdot DBH^{2}}{4\cdot Fc}\right)\cdot H\cdot Ff$$

where *DBH* is diameter at breast height (cm), *H* is total tree height (m), *Fc* a correction factor of units from cm^2^ to m^2^ (10^4^), and *Ff* the average form factor (0.50).

The estimates of the components of variance and genetic parameters were obtained by using the mixed linear models methodology (procedure REML/BLUP - Restricted Maximum Likelihood/Best Linear Unbiased Prediction). The data processing model n° 5 of the software Selegen-REML/BLUP (Resende, 2007) was used to make all the required statistical genetic analyses for the three variables considered in the study. The mathematical modelling allowed obtaining the estimates of the genetic variances between the provenances and the coefficient of determination of the provenance effect, besides those parameters normally predicted when a single population is under evaluation.

The referred model has the following mathematical structure (Resende, 2007):

$$y=X_{r}+Z_{a}+W_{p}+T_{s}+e$$

where *y* is the data vector, *r* is the replicate effect vector (assumed as fixed) added to the overall mean, *a* is the vector of the individual additive genetic effect (assumed as random), *p* is the plot effect vector (assumed as random), *s* is the provenance effect vector (random) and *e* is the vector of experimental errors (random). The capital letters represent the incidence matrices for the cited effects.

Estimates were obtained for the following genetic parameters: heritability coefficient of additive effect (${\hat{h}}_{a}^{2}$), coefficient of determination of plot effect (${\hat{C}}_{plot}^{2}$), coefficient of determination of provenance effect (${\hat{C}}_{prov}^{2}$), additive coefficient of variation [${\hat{CV}}_{a}$(%)], genetic coefficient of variation among progenies [${\hat{CV}}_{\mathrm{gp}}$(%)] and residual coefficient of variation [${\hat{CV}}_{e}$(%)].

The absolute and relative genetic gains were estimated by using the following expressions:

$$\hat{G}s=ds\cdot {\hat{h}}_{a}^{2}$$

where *ds* represents the differential of selection and ${\hat{h}}_{a}^{2}$ the heritability coefficient of additive effects.

And

$$\hat{G}s(\%)=\left(\frac{\hat{G}s}{\bar{X}}\right)\cdot 100$$

where $\hat{G}s$ represents absolute genetic gain and $\bar{X}$ the overall mean of the trait.

Analysis of deviance was also performed for the random effects of the model (progeny, provenance and plot), and the assessment of the statistical significance was made through the Likelihood Ratio Test (LRT).

Finally, the Spearman's correlation coefficient combining DBH, H and Vol was used with the purpose of investigating an eventual modification in the ranking of the selected trees according to their additive values. The formula is as follows:

$$\text{ρ}=1-\left(\frac{6\cdot \sum_{i=1}^{n}di^{2}}{n^{3}-n}\right)$$

where *n* represents the number of observations and *di* ^2^ the square of the positioning differences in the classification between variables.

## Results and Discussion

Taking into account works carried out by the first author in the state of Santa Catarina in 2007 and 2008 (unpublished data), it was possible to make comparisons among the species under study and others planted in the same region with respect to their performances. A sample of the results is shown in Table 2, emphasizing that the all data presented are based on means of selected trees. It can be observed that *E. badjensis* exhibited a better diametric and volumetric growth in Brazil when compared with *E. dunnii*, *E. viminalis* and *E. grandis*, even considering that it had not been submitted to any cycle of breeding so far. Only for tree height performance the species was positioned behind *E. dunnii* and *E. grandis*.

**Table 2:** Mean annual increment (MAI) for DBH, H and Vol obtained from different species of eucalypts planted in the states of Santa Catarina and Rio Grande do Sul in locations geographically close and submitted to similar climatic conditions.

Species	Nr. of trees	Age (years)	Locations	MAI		
				DBH (cm)	H (m)	Vol (m^3^)
*E. dunnii*	32	16 to 17	Irani-SC	2.74	2.44	0.12
*E. viminalis*	20	17	Vargem Bonita-SC, Irani-SC and Campina da Alegria-SC	2.80	1.50	0.14
*E. grandis*	35	17 to 20	Erval Grande-RS and Concórdia-SC	3.09	2.54	0.21
*E. badjensis*	39	17	Campina da Alegria-SC	3.30	2.16	0.27

In the study carried out by Swain and Gardner (2003) in South Africa with the purpose to investigate the performance of subtropical species of eucalypt in regions prone to severe frosts and low temperatures, and even snow and drought, *E. badjensis* came out as a very promising species, representing an alternative to *E. nitens* in climatically critical areas. The authors also mentioned that a breeding programme of *E. badjensis* was established in the 1990s, in which the initial steps were the evaluation of provenances and progenies in a set of environmental conditions in order to evaluate the effects of genotype x site interaction. Comparing the results of ten eucalypt species under study, *E. badjensis* was amongst the most tolerant species to frost, cold, snow, drought, termites and some strains of the fungus *Phytophthora*, the infectious agent of root-rot disease in young trees. The species was declassified only for the level of tolerance to defoliation caused by snout beetle larvae. However, considering the rapid crown recovery, the trouble became less important. The authors also reported the rapid initial growth of the trees for height and diameter.

Swain and Gardner (2003) indicated the species as ideal for growth in South Africa under the following natural condition ranges: mean annual temperature between 14.5 and 18 °C, minimum mean annual precipitation between 800 and 900 mm, altitude between 1,100 and 1,600 m. Such ranges are compatible with the ones found in the location of Campina da Alegria, situated on the plateau of Santa Catarina, where the present experiment of *E. badjensis* was planted.

A preliminary analysis of the growth data allowed getting a series of descriptive statistics for both the complete sample of trees and the selected ones only, and the main results are shown in Table 3. The group of the selected trees was defined in a non-truncated way, because several characteristics were taken into account simultaneously, such as growth rate, sanity and stem straightness. The means for DBH, H and Vol of the selected trees were 56.12 cm; 36.68 m and 4.59 m^3^, respectively, which represents increases around 24%, 10% and 62% compared to the overall means of the trial (45.17 cm, 33.30 m and 2.84 m^3^ for DBH, H and Vol, respectively). These numbers indicated the feasibility of improving the growth rate in a breeding programme, as can be noted when comparing the mean annual increments (MAIs) of the selected trees (3.30 cm, 2.16 m and 0.27 m^3^, for DBH, H and Vol) with those ones of the complete sample (2.66 cm, 1.96 m and 0.17 m^3^, for DBH, H and Vol).

**Table 3:** Results of descriptive statistics and mean annual increment (MAI) obtained for DBH, H and Vol, considering all trees of the experiment and the grouping corresponding to the selected trees (*E. badjensis*, 17 years old, Campina da Alegria-SC).

Attribute	Traits		
	DBH (cm)	H (m)	Vol (m^3^)
	Complete sample (N = 279) / Selected trees (N = 39)		
Mean	45.17 / 56.12	33.30 / 36.68	2.84 / 4.59
Higher value	67.5	42.0	7.01
Lower value	27.7 / 45.8	21.9 / 29.1	0.81 / 2.68
Amplitude	39.8 / 21.7	20.1 / 12.9	6.20 / 4.33
Standard deviation	8.41 / 4.95	3.54 / 2.75	1.25 / 0.99
MAI	2.66 / 3.30	1.96 / 2.16	0.17 / 0.27

The performance of the 39 selected trees is shown in Table 4. The additive genetic value (μ + a) for growth rate (expressed by Vol) was the criterion used for ranking each one. We noted a clear prevalence of individuals from P2 rather than from P1, comprising 35 and 4 trees, respectively. These genotypes belonged to 25 distinctive progenies, which represented around 40% of the total amount of progenies assessed. In theory, this group of trees represents a significant sample of the total variability that could be explored by selection and, as a consequence, minimizes the risks of a relevant level of endogamy in the next generation. The random distribution of the selected trees along the replicates and their respective position within the plots indicated that there was no significant environmental influence on the selection.

**Table 4:** Ranking of the 39 genotypes selected for Vol and genetic parameters estimates for DBH, H and Vol (*E. badjensis*, 17 years old, Campina da Alegria-SC).

Ranking for Vol (m^3^)	Progeny/Provenance ID	DBH (cm)			H (m)			Vol (m^3^)		
		f^1^	a^2^	(μ + a)^3^	f	a	(μ + a)	f	a	(μ + a)
1	20/2	66.2	11.52	56.69	40.2	2.26	35.56	6.92	1.78	4.62
2	01/2	65.9	9.90	55.07	41.1	1.88	35.18	7.01	1.45	4.28
3	20/2	58.9	9.29	54.46	38.4	2.18	35.48	5.23	1.35	4.18
4	32/2	63.3	8.83	54.00	39.3	1.97	35.27	6.19	1.27	4.10
5	19/1	67.5	9.46	54.63	37.2	0.67	33.97	6.65	1.24	4.08
6	37/2	61.1	8.17	53.34	37.5	1.44	34.74	5.50	1.07	3.91
7	09/2	57.0	5.98	51.14	41.1	2.71	36.01	5.24	1.00	3.84
8	17/2	63.0	9.35	54.52	33.0	0.60	33.90	5.15	1.01	3.84
9	18/2	55.7	8.24	53.41	35.4	1.18	34.48	4.31	0.97	3.80
10	20/2	52.2	5.59	50.76	41.1	2.54	35.84	4.40	0.95	3.78
11	08/2	56.7	7.51	52.68	36.3	0.77	34.07	4.58	0.93	3.77
12	19/2	60.5	6.95	52.12	36.9	1.01	34.31	5.30	0.92	3.76
13	14/2	60.5	8.17	53.34	36.3	0.51	33.81	5.21	0.91	3.74
14	07/2	57.3	6.25	51.42	37.8	1.67	34.97	4.87	0.81	3.65
15	19/1	58.9	5.49	50.65	39.0	1.17	34.47	5.31	0.79	3.63
16	40/2	57.0	6.01	51.18	36.9	1.57	34.87	4.70	0.78	3.62
17	18/2	57.6	6.92	52.09	34.2	0.41	33.71	4.46	0.77	3.61
18	34/2	54.7	6.23	51.40	36.3	1.71	35.01	4.27	0.76	3.59
19	05/2	57.9	7.28	52.45	34.8	0.54	33.84	4.59	0.75	3.58
20	31/2	57.0	5.15	50.32	36.9	1.37	34.67	4.70	0.71	3.54
21	19/2	57.3	6.11	51.28	35.1	0.50	33.80	4.52	0.70	3.53
22	19/2	55.7	5.22	50.39	37.8	1.00	34.30	4.61	0.70	3.53
23	19/2	55.1	6.03	51.20	33.6	0.30	33.60	4.00	0.66	3.50
24	09/2	51.9	4.14	49.31	35.4	1.43	34.72	3.74	0.61	3.44
25	27/2	56.7	4.67	49.84	37.8	0.90	34.20	4.77	0.58	3.42
26	32/2	50.9	3.96	49.13	37.2	1.46	34.76	3.79	0.56	3.40
27	04/1	54.1	4.10	49.27	38.1	1.64	34.94	4.38	0.55	3.38
28	04/2	52.2	3.08	48.25	42.0	2.70	36.00	4.49	0.54	3.38
29	38/2	55.4	3.76	48.93	37.8	1.32	34.62	4.55	0.54	3.38
30	37/2	51.9	4.22	49.39	36.3	0.92	34.22	3.84	0.53	3.36
31	37/2	49.3	4.37	49.54	34.8	0.91	34.21	3.33	0.47	3.31
32	11/1	50.9	4.12	49.29	36.0	1.04	34.34	3.67	0.44	3.28
33	26/2	54.1	2.92	48.09	36.9	1.21	34.51	4.24	0.43	3.26
34	35/2	55.7	3.33	48.50	36.6	0.51	33.81	4.46	0.40	3.23
35	11/2	52.8	2.97	48.14	34.5	0.57	33.87	3.78	0.32	3.16
36	27/2	51.9	3.64	48.81	32.1	-0.43	32.87	3.39	0.26	3.09
37	05/2	48.4	3.19	48.36	29.1	-0.94	32.36	2.68	0.19	3.02
38	02/2	49.7	2.97	48.14	30.9	-0.54	32.76	2.99	0.17	3.01
39	11/2	45.8	0.44	45.61	38.7	1.74	35.04	3.19	0.15	2.98
Overall mean (μ)		45.17			33.30			2.84		

Legend:

1 phenotypic value;

2 additive effect;

3 additive value (BLUP) of the mean for the subsequent generation of planting, when growing under similar environmental and silvicultural conditions and assessed at the same age of this experiment.

Another aspect to be considered was the eventual modification in the rankings of the selected trees depending on the traits under comparison. In certain cases, the individual genotypes were not able to keep a nearby position when examining the classification columns. In order to investigate the degree of association between traits according to the rankings, the Spearman's correlation coefficient (ρ) was computed for each pair of variables. The results and respective significance by t-test (considering 30 degrees of freedom presently) were as follows: DBH × H (0.16^ns^), DBH × Vol (0.93**) and, finally, H × Vol (0.41**). This means that DBH and Vol ranked the selected trees in a very similar way. In addition, DBH was the trait that promoted a better indirect representation of the woody production of a tree, corroborating a number of studies carried out in Brazil with eucalypts, both in young trees and adults (Resende *et al*., 1994; Macedo *et al*., 2013; Pinto Júnior JE, 2004, Doctoral Thesis, Universidade Federal do Paraná, Curitiba, PR; Brizolla TF, 2009, Masters Dissertation, Universidade Estadual Paulista “Júlio de Mesquita Filho”, Botucatu, SP; Henriques EP, 2012, Masters Dissertation, Universidade Estadual Paulista “Júlio de Mesquita Filho”, Botucatu, SP).

The contribution of P2 for the new means was evident when examining the results presented in Table 5. For the three traits studied, P2 individually responded by slightly higher values compared with the overall mean (μ).

**Table 5:** Ranking of each population assessed and respective results for genotypic effect (g), genotypic value (μ + g) and new means for DBH, H and Vol (*E. badjensis*, 17 years old, Campina da Alegria, SC).

Ranking	Provenance ID	DBH (cm)				H (m)				Vol (m^3^)			
		g	(μ + g)	Gain	New Mean	g	(μ + g)	Gain	New Mean	g	(μ + g)	Gain	New Mean
1	2	1.41	46.58	1.41	46.58	0.28	33.58	0.28	33.58	0.18	3.01	0.18	3.01
2	1	−1.41	43.76	0.00	45.17	−0.28	33.02	0.00	33.30	−0.18	2.66	0.00	2.84

The results of deviance analysis are shown in Table 6. The LRT revealed the nonexistence of significant statistical differences for the provenance effect in the traits DBH, H and Vol, indicating that the corresponding progenies could be gathered in a single breeding population. On the other hand, the detection of significant differences at progeny level for DBH and Vol showed that there is considerable variation in the performances concerning these traits, a finding that could be reverted to benefits within a breeding programme. The non-significance for the plot effect showed that the compact family block design was effective to control the environmental heterogeneity.

**Table 6:** Summary of the results obtained from the deviance analysis for DBH, H and Vol (*E. badjensis*, 17 years old, Campina da Alegria-SC).

Effect	Deviance			LRT^1^			Variance component			Coefficient of determination (C^2^)		
	DBH	H	Vol	DBH	H	Vol	DBH	H	Vol	DBH	H	Vol
Progeny	1,437.36	971.57	410.86	5.83*	1.88^ns^	4.11*	72.973**	12.437**	1.5775^ns^	0.443	0.312	0.358
Provenance	1,434.20	970.24	408.74	2.67^ns^	0.55^ns^	1.99^ns^	4.840*	0.261^ns^	0.081^ns^	0.066	0.021	0.051
Plot	1,431.62	970.93	406.92	0.09^ns^	1.24^ns^	0.17^ns^	2.888^ns^	1.941^ns^	0.077^ns^	0.041	0.158	0.050
Error	-	-	-	-	-	-	-	-	-	0.449	0.509	0.540
Complete model	1,431.53	969.69	406.75							1.000	1.000	1.000

1 Likelihood Ratio Test;

** Significant at the level of 1% of probability in the X^2^ (chi-square) test;

* Significant at the level of 5% of probability in the X^2^ test;

ns Not significant in the X^2^ test.

Significances of X^2^ test: 3.84 (5%) and 6.63 (1%).

Interestingly, the same *Badja S.F*. provenance, inclusively with the same amount of progenies, was also included in provenance/progeny trials in South Africa, and Swain and Gardner (2003) reported the superiority of this material for growth at 5.5 years of age in comparison with the provenance named *Brown Mountain* via *Nimmitabel*, New South Wales, Australia (latitude 36°29′ S, longitude 149°19′ E, altitude 1,100 m), in this case represented by 20 progenies. Even though these results were obtained at very early ages, the *Badja S.F*. provenance proved to be of great importance to develop a breeding programme, both in South Africa and in Brazil.

The estimates of genetic parameters are summarized in Table 7, which presents the values of the variances and the coefficients that are particularly useful to evaluate the potential of the species in supporting a breeding programme.

**Table 7:** Summary of the results obtained from the statistical genetic analysis for DBH, H and Vol (*E. badjensis*, 17 years old, Campina da Alegria-SC).

Parameters of the genetic evaluation (N^1^ = 279)		Traits		
		DBH (cm)	H (m)	Vol (m^3^)
$\sigma_{a}^{2}$	Additive variance	32.340	3.876	0.565
$\sigma_{plot}^{2}$	Environmental variance among plots	2.841	1.967	0.075
$\sigma_{prov}^{2}$	Genotypic variance between provenances	4.837	0.264	0.080
$\sigma_{e}^{2}$	Residual variance	32.955	6.330	0.857
$\sigma_{ph}^{2}$	Phenotypic variance at individual level	72.973	12.437	1.577
$h_{a}^{2}$	Heritability coefficient of additive effect	0.443 ± 0.226	0.312 ± 0.189	0.358 ± 0.203
$C_{plot}^{2}$	Coefficient of determination of plot effect	0.039	0.158	0.048
$C_{prov}^{2}$	Coefficient of determination of provenance effect	0.066	0.021	0.051
*CV_a_*(%)	Additive coefficient of variation	12.590	5.912	26.511
*CV_gp_*(%)	Genetic coefficient of variation among progenies	6.295	2.956	13.255
*CV_e_*(%)	Residual coefficient of variation	7.788	5.624	18.947
Overall mean		45.17	33.30	2.84

1 sample size.

The results show the feasibility to reach considerable genetic gains, despite the relatively large confidence interval for ${\hat{h}}_{a}^{2}$ (heritability coefficient of additive effect). According to Garcia (1989) and Pimentel-Gomes and Garcia (2002), the residual coefficients of variation [${\hat{CV}}_{e}$(%)] can be considered of low magnitude for all traits, indicating an acceptable environmental control and satisfactory accuracy of the genetic parameter estimates. The additive coefficient of variation [${\hat{CV}}_{a}$(%)] and the descriptive statistics found for the traits assessed (Table 3) corroborate the good perspectives of the provenances under study for growth gains. It is worthy of note that these materials brought to Brazil were not submitted to any previous genetic selection in Australia due to the fact that the seeds were collected originally from trees growing in natural stands.

The expected genetic gains [$\hat{G}s$ and $\hat{G}s$(%)] when using seedlings obtained through the recombination of the 39 selected trees are shown in Table 8. By examining the results, it is possible to deduce that there is an effective opportunity to increase the growth rates for DBH and Vol, as long as the new improved plantations are established under very similar environmental conditions and silvicultural techniques. In studies made by Swain and Gardner (2003) with *E. badjensis* in South Africa, genetic gains around 11 and 12% over the experiment mean were reported for DBH at the age of 5.5 years when selecting the best 15 progenies of the trial, which are comparable with the expected gain.

**Table 8:** Original means of P1/P2, absolute and relative genetic gains and the expected means of the improved P1/P2 for DBH, H and Vol (*E. badjensis*, 17 years old, Campina da Alegria-SC).

Attribute	Traits		
	DBH	H	Vol
Mean of the original provenances (P1/P2)	45.17 cm	33.30 m	2.84 m^3^
Mean of selected trees	56.12 cm	36.68 m	4.59 m^3^
ds	10.95 cm	3.38 m	1.75
${\hat{h}}_{a}^{2}$	0.443	0.312	0.358
${\hat{G}}_{s}$	4.85 cm	1.05 m	0.63 m^3^
${\hat{G}}_{s}$(%)	10.74	3.17	22.06
Mean of improved provenances (P1/P2)	50.02 cm	34.35 m	3.47 m^3^

Based on the results of the present work, we conclude that the genetic variability of the species is appropriate for carrying out a breeding programme for increasing the growth rate in successive cycles of improvement, especially for the traits DBH and Vol. Additionally, for the three traits evaluated (DBH, H and Vol), the statistical differences in performance between the two provenances were not significant but, at the progeny level, there were significant differences for DBH and Vol. Finally, the level of correspondence between the rankings of the selected trees when comparing the traits DBH and Vol was very high, indicating that the first could be used as an indirect trait for selection focused on the increase in the volumetric growth rate as a whole.
